# Recombinant Factor C as an In Vitro Assay for the Residual Pathogenicity Evaluation of Veterinary Autogenous Vaccines

**DOI:** 10.3390/vetsci11120673

**Published:** 2024-12-21

**Authors:** Antonella Di Paolo, Rosario Liberti, Lucia Anzalone, Claudia Colabella, Andrea Felici, Giulio Severi, Monica Cagiola

**Affiliations:** 1 Istituto Zooprofilattico Sperimentale Dell’Umbria e Delle Marche “Togo Rosati”, via G. Salvemini 1, 06126 Perugia, Italy; 2 Laboratorio Analisi “Ospedale S. Matteo Degli Infermi”, USL Umbria 2, Via Loreto, 3, 06049 Spoleto, Italy

**Keywords:** bacterial endotoxin testing, recombinant factor C, LAL test, veterinary autogenous vaccine

## Abstract

Background: Veterinary autogenous vaccines, similar to all injectable pharmaceutical products, must be tested to assess endotoxin concentrations. The Limulus Amebocyte Lysate Test (LAL test) is widely used in in vitro quality control assays for endotoxin detection, although it presents some ethical issues related to the production of reagents and is also characterized by a low specificity due to other contaminants that can activate the reaction. For all these reasons, a new recombinant factor C LAL test was developed. Aim: In this study, we described the comparison between two LAL test methods for in vitro quality control of veterinary autogenous vaccines, with the aim of evaluating the most suitable method and establishing an endotoxin concentration range for two different matrices. Methods: Two hundred batches of two different vaccine matrices were tested using the kinetic chromogenic LAL test and recombinant factor C endotoxin detection assay commercial kits. Results and Conclusions: Statistical analysis conducted after the validation of the recombinant factor C test exhibited a statistically significant correlation between the two methods and for both vaccine matrices, suggesting that the animal-free assay can be used as a routine quality control test for veterinary autogenous vaccines.

## Simple Summary

Testing active parenteral injection molecules and medical devices for pyrogens (fever-inducing substances) is critical for quality control assurance and end users’ safety. The original rabbit pyrogen test has largely been replaced by different endotoxin tests that are Limulus Amebocyte Lysate (LAL)-based. Since 2004, a bacterial endotoxin assay with a recombinant factor C (rFC), the endotoxin reporter protein of LAL, has been adopted as an animal-free alternative to conventional LAL assays. Likewise, many studies compared LAL and rFC on different injectables but never on complex matrices such as veterinary autogenous vaccines. Here, we compare two bacterial endotoxin testing methods and demonstrate that rFC is equivalent and comparable to chromogenic kinetic LAL assays for the residual pathogenicity assessment of veterinary autogenous vaccines.

## 1. Introduction

All injectable drugs, such as veterinary vaccines, and other parenteral pharmaceutical products must be tested for quality assurance before release, including pyrogen detection [1,2].

Pyrogens, as well-studied Gram-negative lipopolysaccharides (LPSs), stimulate macrophages and activate inflammatory pathways with the release of cytokines, causing uncontrolled, life-threatening inflammatory reactions [3,4,5].

Different tests have been used in accordance with the need to research endotoxin contamination in all injectable pharmaceutical preparations, such as the rabbit pyrogen test (RPT) and the modern bacterial endotoxin test (BET) [1,2,3].

The first and best-known BET is the Limulus Amebocyte Lysate test (LAL test), which measures the coagulation of lysed amebocytes caused by endotoxins in horseshoe crabs’ blood cells.

Small amounts of LPSs activate the first of two serine-zymogen proteases, factor C; this initiates the cascade activation of factor B; the active form of the latter promotes the activation of the last enzyme involved in the coagulation cascade, which is the proclotting enzyme that causes the coagulation [6,7].

However, other unspecific substances, such as beta glucans, can determine the activation of an alternative coagulation cascade in which a different serine-zymogen protease, factor G, directly activates the proclotting enzyme; this results in coagulation. This alternative pathway may interfere with the LAL test, resulting in a reduction in specificity [1,8,9].

In addition, the LAL test could exhibit ethical issues due to the intensive use of horseshoe crabs in the production of the necessary reagents, resulting in a drastic reduction in horseshoe crab populations to the extent that the International Union for Conservation of Nature declared this species as a “vulnerable population” [10,11,12,13,14].

For all these reasons, a laboratory non-animal-derived recombinant factor C (rFC) reagent was manufactured and commercialized in a new format kit from many companies.

rFC is characterized by its higher affinity and sensitivity to LPSs compared to standard LAL test methods, resulting in an assay’s increased specificity [1,15,16].

To date, several commercial rFC LAL assays are available, and the *European Pharmacopoeia* has listed the new LAL format as a compendial method [17,18,19].

Many validations have been performed on different pharmaceutical molecules, even with respect to human and veterinary commercial vaccines [1,2,20], but information about their suitability for use with the BET method on autogenous vaccines is still lacking.

Autogenous vaccines (AVs) are a peculiar category of veterinary vaccines prepared from inactivated bacterial or viral cultures isolated from infected animals; this specific custom-made product is adopted with the aim of immunizing animals against circulating pathogens during outbreaks or when a commercial vaccine is not available.

In Italy, since 1994, AV production and commercialization have been permitted only for Istituti Zooprofilattici Sperimentali (IIZZSS) under the authorization of the Italian Ministry of Health (D.M. n. 287 art.3 paragraph 1).

Even if it is an emergency production procedure, AVs have to be tested for residual pathogenicity in laboratory animals (mice/guinea pigs) before release, similar to all other vaccines.

As stated in updated Italian and European laws (Regulation EU 2019/6, European Directive n. 63/2010, and Italian Legislative Decree n.26/2014) concerning the involvement of laboratory animals in the quality control (QC) process, AVs were equalized to commercial ones and controlled with in vitro tests to replace in vivo assays [21,22,23,24].

These assays must be carried out to check for residual pathogenicity, in addition to sterility control; moreover, chemical analyses should be carried out for the detection of inactivating substance levels and low-grade formaldehyde.

Moreover, depending on the nature of the starting culture for AVs, residual pathogenicity can be assessed using different in vitro tests.

In fact, while Gram-positive and viral AVs are tested with viability cell-based assays, i.e., MTS/MTT or ATP and/or the monocyte activation test, Gram-negative matrices are tested at our laboratory with BET methods.

Notably, the application of BET methods to AVs is used to quantify endotoxin content—in vaccine batches—not as contaminants but as a peculiar feature of the releasing lot.

In addition, with respect to the 3Rs philosophy, the rFC LAL test’s adoption represents a valid alternative to the animal-based LAL assay for QC.

In this study, we describe a comparison between the chromogenic kinetic LAL test (KCA) and the recombinant factor C LAL (rFC) endotoxin assay test in complex matrices, such as autogenous vaccines prepared at the Pharmaceutical Department of Istituto Zooprofilattico Sperimentale dell’Umbria e delle Marche “Togo Rosati” (IZSUM) in Perugia, Italy.

## 2. Materials and Methods

### 2.1. Veterinary Autogenous Vaccines (AVs)

BET assays were carried out on two vaccine matrices produced as previously described [21,25]. Briefly, a total of 100 *Escherichia coli* and 100 *Pasteurella* spp. strains were collected at the Pharmaceutical Department (IZSUM) and processed for emergency custom-made formulations at different farms during 2023 and 2024.

After characterization was carried out, a single pure isolated colony of each pathogen was amplified in the proper culture medium (Minca Medium for *E. coli* and Brucella broth for *Pasteurella* spp., respectively) produced at the “Culture Media unit “of IZSUM; these were adjusted to the final concentration (1 × 10^9^ CFU/mL), chemically inactivated via a formalin solution (0.04%), and then adjuvanted with a solution of alum hydroxide (20% *v*/*v*) [23,24].

Every lot produced was tested for quality control assurance before BET, including neutral pH determination, low-grade formaldehyde-free assessment via chemical analysis, and finally, strain purity assessment and inactivation.

### 2.2. Kinetic Chromogenic LAL Assay

The Kinetic-QCL™ Kinetic Chromogenic LAL Assay (KCA) (Catalog #: 50-650U) was purchased from Lonza (Basel, Switzerland); assays were carried out following the manufacturer’s instructions.

All reagents and samples were reconstituted and diluted, adopting only endotoxin-free water from the purchased kit.

Briefly, the control standard endotoxin (CSE, *Escherichia coli* O55:B5, stated 50 EU/mL) was rehydrated under continuous stirring for 15 min. The reconstituted standard was immediately used to generate a standard curve from 50 to 0.005 EU/mL.

AVs were diluted 1:10,000, and 100 µL was added to a 96-well flat plate in quadruplicate (two wells for endotoxin quantification and two wells for spike recovery, adding a final CSE concentration of 5 EU/mL). Endotoxin-free water was assessed as a negative control.

The plate was then pre-incubated at 37 °C for at least 10 min. Close to the end of the pre-incubation period, the lysate was reconstituted in the dark and immediately added to the plate.

The analysis was performed at 37 °C (40 consecutive reads every 150 s) using a BioTek Synergy HTX multi-mode reader equipped with Gen5 software, version 3.

### 2.3. Recombinant Factor C LAL Assay

The ENDONEXT™ EndoZyme^®^ II Recombinant Factor C (rFC) Endotoxin Detection Assay (890031) was obtained from bioMèrieux (Marcy-l’Etoile, France).

Briefly, the CSE (*Escherichia coli* O55:B5 stated 50 EU/mL) was rehydrated under continuous stirring for 10 min. The reconstituted standard was immediately used to generate a standard curve from 50 to 0.005 EU/mL.

AVs were diluted 1:50,000, and 100 µL was added to a 96-well flat plate in quadruplicate (two wells for the endotoxin quantification and the other two for spike recovery, adding a final CSE concentration of 0.5 Eu/mL). Endotoxin-free water was included as the negative control.

The assays were performed according to the manufacturer’s instructions using a BioTek Synergy HTX multi-mode reader equipped with Gen5 software. Samples were pre-incubated at 37 °C and combined with 100 µL of assay reagents (obtained by mixing substrates, enzymes, and assay buffers).

Fluorescence reading was performed with excitation/emission set at 380/445 nm, and 15 s of shaking at medium intensity took place before the reading was carried out at time point 0.

### 2.4. Assay Validation

Linearity, accuracy, precision, and robustness and the respective acceptance criteria (AC) previously determined for KCA were applied for the rFC validation assay in the detection of endotoxins, following the guidelines for the methods’ validation reported in Chapters 5.26 and 5.27 of the *European Pharmacopoeia* [26,27].

Briefly, the CSE provided in the kit was adopted to generate standard curves, as described above.

A BET was carried out using four sample replicates; two replicates were spiked to assess potential interferences via the addition of positive product controls (5 EU/mL for KCA and 0.5 EU/mL for rFC of CSE, respectively).

Linearity AC exhibited a correlation coefficient (|R|) greater than 0.9800 for the standard curve.

Accuracy AC exhibited sample recovery between 50% and 200%.

Precision and robustness ACs exhibited a coefficient of variation (CV) obtained from repeated measurements of an assay (repeatability). Analyses (intermediate precision) and laboratories (reproducibility) were tested for precision. The CV (%) of both the sample and PPC recovery was <25% for robustness.

Moreover, the KCA and rFC LAL methods were compared. For each assay, a fresh standard curve with five scalar concentrations of CSE diluted with endotoxin-free water was prepared.

### 2.5. Endotoxin Quantification in AVs

All lots were tested in quadruplicate: two replicates for the endotoxin load and two replicates for PCC recovery (%); moreover, differences (expressed as %) were calculated for each reagent.

Samples were diluted in endotoxin-free water at a final concentration of 1:50,000 for rFC and 1:10,000 for the KCA assay; this was carried out based on previous inhibition/enhancement tests and did not exceed the maximum valid dilution ratio. CSE was added to positive product controls as described above. The endotoxin load and recovery were calculated with a 4-parameter logistic curve.

### 2.6. Statistical Analysis

Descriptive analyses were conducted to establish reference ranges according to the guidelines outlined in the “Guidelines for the determination of reference intervals (RIs) in veterinary species” by the ASVCP (American Society for Veterinary Clinical Pathology).

Calculations were carried out in STATA 16.1 (StataCorp LLC, 4905 Lakeway Drive College Station, Texas, USA) and Microsoft Excel 2013 using Reference Value Advisor V2.1 [28].

All batches of *Escherichia coli* and *Pasteurella* spp. were analyzed in parallel with the two methods. The normality of the distribution and the presence of outliers were assessed (with the Anderson–Darling test and Tukey’s method, respectively), with outliers appropriately removed from further analysis.

The nonparametric method was applied to determine the reference intervals. This was achieved by calculating the central 95% range of the collected data, which involves excluding the lowest and highest 2.5% of values to identify the central distribution accurately.

The association between the KCA and rFC tests (for both *Escherichia coli* and *Pasteurella* spp.) was determined using Spearman’s rank correlation coefficient (ρ).

## 3. Results

### 3.1. Validation of rFC LAL Assay

Linearity was analyzed based on CSE concentration results, adopting a standard curve calculated using a regression model [fitting a linear model log(Y) = Alog(X) + B].

The |R| values of assays 0.9999 and/or 1.000 met the acceptance criterion (|R| ≥ 0.980).

For accuracy, three sets of duplicate sample concentrations were prepared, and three assays were independently conducted by a single technician.

The endotoxin loads in the AVs were compared with the expected CSE concentrations. All samples fell within the acceptable range of 50–200%.

To achieve precise results, three analysts performed each of the three assays, with three quadruplicate samples prepared for each assay.

The average endotoxin concentration and PPC recovery (%) were calculated for each assay.

All CV results (%) met the acceptance criterion of <25% for both actual endotoxin concentrations and positive product control recovery.

The CV of the actual endotoxin concentration (%) and positive product control recovery (%) obtained from three different reagent lots was adopted to assess robustness.

All CV (%) results met the acceptance criteria of <25%.

### 3.2. Endotoxin Quantification in AVs

All AVs were assessed in parallel with KCA and rFC LAL assay methods; endotoxin quantification and spike recovery, expressed as EU/mL, were recorded as mean values for statistical analyses.

### 3.3. Statistical Analysis

**Figure 1:**
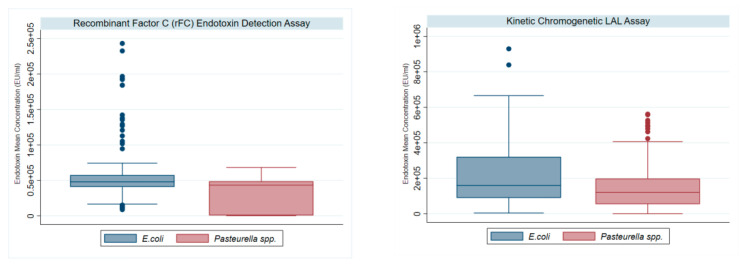
Mean endotoxin concentration for KCA and rFC LAL assays. The distribution of the mean endotoxin concentrations of all two hundred samples obtained with KCA and rFC LAL assay methods is represented using a box plot.

The Spearman’s correlation index reflected the presence of a significant correlation between the two variables for both *Escherichia coli* (ρ = 0.341; *p*-value < 0.001) and *Pasteurella* spp. (ρ = 0.346; *p*-value < 0.01) (Table 1) (Figure 1).

## 4. Discussions

The development of recombinant factor C LAL assays and the subsequent recognition in the *Pharmacopoeia* as a compendial method for the detection and quantification of endotoxins in injectable pharmaceutical products represented a major step forward in the attempt to overcome excessive horseshoe crab use in the production of classical LAL assay reagents [18,19]. In addition, non-animal-derived factor C allowed for a marked increase in the specificity of the test by excluding false-positive results related to contaminants, which are responsible for the activation of the coagulation cascade in conventional LAL assays [1,8].

The *European Pharmacopoeia* listed the LAL methods among the tests for evaluating the residual pathogenicity of veterinary vaccines, including autogenous vaccines. However, although different rFC assay validations have been reported for multiple human or veterinary drugs, there are no reports about the validation of the same method for autogenous vaccines.

In our laboratory, since 2021, we adopted KCA for routine Gram-negative vaccine matrix control before releasing batches, replacing in vivo abnormal toxicity tests (performed on five mice per lot) after 5 years of consistent quality assurance, saving at least 800 mice per year.

For this reason, here, we evaluated the possible application of rFC as an alternative method through a validation process and established that all the parameters considered (linearity, accuracy, precision, and robustness) agreed with acceptance criteria according to Ph. Eur. Chapter 5.2.14. [26,27,29].

For these reasons, we adopted standard manufacturing and control processes, acquiring standard certified reactants (included in the kit and already tested and normalized for the analysis output) and referring to analyzed matrix features.

Consequently, we focused on comparing two BET methods using two different bacterial vaccine matrices for a total of two hundred batches produced in our laboratory between 2023 and 2024 upon request.

We conducted tests with both methods in parallel; however, because of the complexity of the matrix used and the higher susceptibility of the rFC assay, it was necessary to dilute the samples at 1:50,000 compared with the 1:10,000 dilution for KCA [10].

The general analysis of the results for independent samples with both methods met the AC; moreover, the rFC LAL method exhibited better endotoxin recovery compared to KCA.

In addition, we performed statistical analysis. We first assessed a reference range for each AV matrix for both methods and highlighted that all samples met a reference range with a coefficient of variation of <1.

In addition, we observed a reduction in the mean value of endotoxin concentrations (considering the dilution factor) between the two methods.

These results are in line with the rFC format’s advantage for LPS detection compared to KCA. Thanks to its higher specificity, all possible interfering substances that could activate factor C and that may be found in a complex matrix such as AVs were excluded from quantification.

Furthermore, by calculating the Spearman’s correlation index, we observed that the two methods were not only correlated with respect to both matrices but also that *Pasteurella* spp. lots were also better distributed via both methods compared to the *E. coli* samples.

This difference in value distribution is probably due to the non-proportional correlation of LPS molecules in bacterial walls and the number of CFUs in the culture media.

Moreover, we assumed that the *Pasteurella* spp. matrix exhibited a more normal distribution due to the growing medium and microenvironment conditions, in addition to the specific traits of different single strains processed simultaneously.

## 5. Conclusions

In conclusion, the data presented in this study suggest that recombinant factor C LAL represents a valid alternative animal-based LAL assay for evaluating the residual pathogenicity of complex vaccine matrices, such as veterinary autogenous vaccines.

Moreover, the reference interval calculated represents an important dataset for the routine application of these methods at our laboratory, as it is used to estimate the endotoxin load in Gram-negative AVs, as reported in the *European Pharmacopoeia*.

The endotoxin concentration in the released lot could be useful in veterinary practice, helping veterinarians in deciding the suitable dose for inoculating animals in contingent situations.

Even if the dataset is not large, it provides a significant step that advances the application of the 3R philosophy in veterinary science and contributes to the spread and use of in vitro methods to save animals.

## Figures and Tables

**Table 1:** Descriptive test results and reference intervals (LRL: lower; URL: upper) with their 90% confidence interval (90% CI). Distribution: non-Gaussian. Method: nonparametric.

AV	Method	N	Mean	Median	SD	CV	LRL (90% CI)	URL (90% CI)	Spearman’s Correlation Index (ρ)
*Escherichia* *coli*	KCA	97 ^a^	226,870	159,070	191,253	0.79	16,448.452 (4170–40,904)	760,703.25 (40,904–582,589)	0.341
	rFC	99 ^b^	59,861	47,975	49,902	0.83	10,950 (8850–11,548)	214,587.5 (11,547.5–188,251)	
*Pasteurella* spp.	KCA	98	154,655	120,113	145,705	0.94	180 (65–218)	542,991.25 (218–495,169)	0.346
	rFC	98	33,761	43,325	21,723	0.64	250 (250–250)	59,361.875 (250–56,333)	

Excluded outlier values: a: 1,074,865–1,289,245–1,409,190; b: 332,300.

## Data Availability

The datasets generated and analyzed during the current study are available upon request from the corresponding author, A.D.P.
